# A novel transcription factor-based signature to predict prognosis and therapeutic response of hepatocellular carcinoma

**DOI:** 10.3389/fgene.2022.1068837

**Published:** 2023-01-04

**Authors:** Yanbing Yang, Xuenian Ye, Haibin Zhang, Zhaowang Lin, Min Fang, Jian Wang, Yuyan Yu, Xuwen Hua, Hongxuan Huang, Weifeng Xu, Ling Liu, Zhan Lin

**Affiliations:** ^1^ Department of Radiology, Mengchao Hepatobiliary Hospital of Fujian Medical University, Fuzhou, China; ^2^ Department of Orthopedics, Dongguan People’s Hospital, Dongguan, China; ^3^ Department of Medical Oncology, The Affiliated Cancer Hospital of Zhengzhou University, Zhengzhou, China; ^4^ Department of Radiology, The First Affiliated Hospital of Dali University, Dali, China

**Keywords:** transcription factor, high mobility group AT-hook protein 1, MAF BZIP transcription factor G, prognosis, therapeutic response, hepatocellular carcinoma

## Abstract

**Background:** Hepatocellular carcinoma (HCC) is one of the most common aggressive malignancies with increasing incidence worldwide. The oncogenic roles of transcription factors (TFs) were increasingly recognized in various cancers. This study aimed to develop a predicting signature based on TFs for the prognosis and treatment of HCC.

**Methods:** Differentially expressed TFs were screened from data in the TCGA-LIHC and ICGC-LIRI-JP cohorts. Univariate and multivariate Cox regression analyses were applied to establish a TF-based prognostic signature. The receiver operating characteristic (ROC) curve was used to assess the predictive efficacy of the signature. Subsequently, correlations of the risk model with clinical features and treatment response in HCC were also analyzed. The TF target genes underwent Gene Ontology (GO) function and Kyoto Encyclopedia of Genes and Genomes (KEGG) pathway enrichment analyses, followed by protein-protein-interaction (PPI) analysis.

**Results:** A total of 25 differentially expressed TFs were screened, 16 of which were related to the prognosis of HCC in the TCGA-LIHC cohort. A 2-TF risk signature, comprising high mobility group AT-hook protein 1 (HMGA1) and MAF BZIP transcription factor G (MAFG), was constructed and validated to negatively related to the overall survival (OS) of HCC. The ROC curve showed good predictive efficiencies of the risk score regarding 1-year, 2-year and 3-year OS (mostly AUC >0.60). Additionally, the risk score independently predicted OS for HCC patients both in the training cohort of TCGA-LIHC dataset (HR = 2.498, *p* = 0.007) and in the testing cohort of ICGC-LIRI-JP dataset (HR = 5.411, *p* < 0.001). The risk score was also positively correlated to progressive characteristics regarding tumor grade, TNM stage and tumor invasion. Patients with a high-risk score were more resistant to transarterial chemoembolization (TACE) treatment and agents of lapatinib and erlotinib, but sensitive to chemotherapeutics. Further enrichment and PPI analyses demonstrated that the 2-TF signature distinguished tumors into 2 clusters with proliferative and metabolic features, with the hub genes belonging to the former cluster.

**Conclusion:** Our study identified a 2-TF prognostic signature that indicated tumor heterogeneity with different clinical features and treatment preference, which help optimal therapeutic strategy and improved survival for HCC patients.

## Introduction

Hepatocellular carcinoma (HCC), which comprises 75%–85% of all cases with primary liver cancer, ranks sixth regarding incidence among malignant cancers and is the third leading cause of cancer-related mortality worldwide (Sung et al., 2021). Currently, less than 20% patients with this disease survive over 5 years in China (Allemani et al., 2018). Even for patients with early diagnosis and surgical resection-included multidisciplinary cancer management, the long-term prognosis remains unfavorable, with over 800,000 deaths owing to HCC (Sung et al., 2021). Considering the limited predictive efficacy of conventional models, it is an urgent need to develop novel predictive tools with promising prognostic biomarkers and therapeutic targets to improve the clinical outcome of HCC patients.

Transcription factors (TFs) are regulatory DNA-binding proteins expressed in human cells which play vital roles for signal transduction. The DNA binding domains of TFs recognize specific DNA sequences in the promoter or enhancer regions to regulate target gene transcription (Lambert et al., 2018). TFs are tightly involved in the signaling pathways of cells, with the deregulation of few ones inducing profound influences on multiple gene activities. Recent studies have underlined the contributions of TFs to the malignant behaviors of cancer, including initiation, progression, invasion, metastasis, as well as chemoresistance (Vishnoi et al., 2020). Chronic hepatitis B virus (HBV) infection may accelerate liver fibrosis and cirrhosis and eventually induce hepatocarcinogenesis. According to Turton et al., there are host TFs, both ubiquitous and liver-enriched, participating in the HBV replication process. Moreover, disrupting the binding ability of those TFs would aid with viral eradication for patients (Turton et al., 2020). A recent study summarized the oncogenic features of the sex determining region Y-related high-mobility group box (SOX) TFs in HCC and found their close associations with numerous immune cells and immune-related molecules in HCC (Luo et al., 2022). Also, another TF of pyruvate kinase M2 isoform (PKM2) was reported to induce monocyte-to-macrophage differentiation, leading to tumor microenvironment remodeling and HCC progression (Hou et al., 2020). Sorafenib, a tyrosine kinase inhibitor, is an FDA-approved first-line drug for advanced HCC. Transcription factors YAP/TAZ were found as pivotal drivers of sorafenib resistance in HCC by repressing sorafenib-induced ferroptosis (Gao et al., 2021).

Despite that accumulating reports support the roles of individual TFs for the malignancy of HCC, effective predictive models based on TFs for the prognosis and treatment response are lack for HCC patients to aid clinical decision. In this study, we aimed to systematically investigate the abnormally expressed TFs in HCC and uncover a TF-based signature able to predict prognosis and therapeutic efficacy for patients based on multiple public databases.

## Materials and methods

### Data collection

The transcriptomic data of TCGA-LIHC dataset and clinical information of 371 HCC patients and 50 non-tumor samples were obtained from The Cancer Genome Atlas (TCGA) data portal up to 22 July 2022 (https://portal.gdc.cancer.gov/repository). The raw count data were normalized by the “limma” R package. The transcriptomic data and clinical information of ICGC-LIRI-JP dataset were downloaded from the HCCDB database (http://lifeome.net/database/hccdb/download.html), which included 212 HCC tumor samples and 177 non-tumor samples (Lian et al., 2018). Normalized read count values were used. Additionally, a third transcriptomic data and clinical information of GSE116174 dataset including 64 HCC patients were downloaded from the Gene Expression Omnibus (GEO) (https://www.ncbi.nlm.nih.gov/gds/). The TCGA cohort was used as the training set, and the ICGC and GSE116174 cohorts were as the validation sets. Another GEO cohort of GSE104580 was obtained to investigate the signature association with transarterial chemoembolization (TACE) treatment. A total of 1,639 TFs reported by Lambert et al. were included for signature establishment (Lambert et al., 2018). Since all the data were publicly available, it was not required for additional ethical approval.

### Differentially expressed TFs (DETFs)

Differentially expressed genes (DEGs) were identified by the “limma” R package, with |log2FC| > 1 and an adjusted *p*-value <0.05 in the TCGA and ICGC cohorts. Those overlapped with the TF list were DETFs, which was visualized by a Venn diagram drawn by an online tool (https://bioinformatics.psb.ugent.be/webtools/Venn/). The expression profile of DETFs in the TCGA cohort was plotted using the “pheatmap” R package.

### Construction and validation of the TF-related risk score

The prognostic significance of individual DETFs was investigated by univariate Cox analysis based on the TCGA cohort, where those with follow-up time more than 0 day being included. The correlation between prognosis-related TFs was further analyzed by the “corrr” R package. Then, the Least Absolute Shrinkage and Selection Operator (LASSO) penalized Cox proportional hazards regression was applied to construct an optimal model *via* the “glmnet” R package (Friedman et al., 2010). TFs with non-zero coefficients were selected with 10-fold cross-validation. The risk score of each patient was determined by the following formula: risk score = (expression level of TF 1×coefficient) + (expression level of TF 2 × coefficient) + … + (expression level of TF n × coefficient). The patients were classified into high-risk and low-risk groups according to the optimal threshold of the risk score, which was determined by the “survminer” R package. To estimate the predictive accuracy of the TF signature, the area under the curve (AUC) value of receiver operating characteristic (ROC) curve was calculated *via* the “survivalROC” R package. Univariate followed by multivariate Cox regression analyses were performed to determine the independence of TF signature for the prognosis of patients using the R package “survival”, which was plotted by the R package of “forestplot”. Subsequently, the predictive value of TF signature was further confirmed in the validation cohorts of ICGC-LIRI-JP and GSE116174.

### Response evaluation of therapeutic agents

To infer the potential therapeutic agents, the “pRRophetic” package in R was used to predict the half-maximal inhibitory concentration (IC_50_) of numerous drugs for HCC patients based on the TCGA and ICGC cohorts, by constructing the ridge regression model according to the expression profile of TF signature and the cell line expression spectrum in the Genomics of Drug Sensitivity in Cancer (GDSC) (www.cancerrxgene.org/) (Geeleher et al., 2014).

### Functional enrichment analysis

The DEGs between the high- and low-risk groups were identified by the “limma” R package with |log2FC| ≥ 1 and an adjusted *p*-value <0.05. Then, the DEGs underwent Gene Ontology (GO) and Kyoto Encyclopedia of Genes and Genomes (KEGG) analyses using the “clusterProfiler” R package, where adjusted *p* values with the BH method were used.

### Protein-protein interaction

The common DEGs between groups in both the TCGA and ICGC cohorts were uploaded to the STRING online database (https://string-db.org/) to generate a protein-protein interaction (PPI) network. Those with an interaction score above 0.4 were selected. The PPI network was visualized by the Cytoscape software (version 3.9.1). The MCODE plug-in was used to screen functional modules. The top 10 hub genes were selected by the MCC method *via* the cytoHubba plug-in. Additionally, the interactions between the hub genes were validated by the “corrplot” R package.

### Reverse transcription quantitative polymerase chain reaction (RT-qPCR)

Five patients with primary HCC were retrospectively collected at the Mengchao Hepatobiliary Hospital of Fujian Medical University, China. Formalin-fixed and paraffin-embedded (FFPE) tissues from tumor and adjacent non-tumorous specimens were obtained to determine the transcriptional expressions of TFs in the signature. The study protocol was approved by the Ethics Committee of our institution. All patients provided informed consent. Total RNA of the FFPE tissues was isolated by using the FFPE RNA Kit (AmoyDx, China), and subjected to reverse transcription using the FastKing-RT SuperMix (Tiangen, Beijing, China). Gene expression was then assessed by RT-qPCR using the GoTaq qPCR Master Mix (Promega, WI, United States) and the ABI 7500 Real-Time PCR system (Thermo Fisher Scientific Inc., MA, United States), with GAPDH used as a housekeeping gene. The temperature protocol consisted of 95°C for 1 min for initial denaturation followed by 45 cycles at 95°C for 10 s and at 60°C for 30 s. The relative expression levels were calculated using the 2^−ΔΔCT^ method and normalized by GAPDH. The primer sequences were as follows: HMGA1 forward: 5′-AAG​GGG​CAG​ACC​CAA​AAA-3′, reverse: 5′-TCC​AGT​CCC​AGA​AGG​AAG​C-3’; MAFG forward: 5′-TCT​AGG​GCT​TGG​GCT​GAT​CT-3′, reverse: 5′-TGT​AGC​CCT​TGT​CTG​CAC​TG-3’; GAPDH forward: 5′- CAG​GAG​GCA​TTG​CTG​ATG​AT-3′, reverse: 5′-GAA​GGC​TGG​GGC​TCA​TTT-3’.

### Immunohistochemistry (IHC)

The FFPE blocks of tumor and adjacent tissues were used for IHC staining by applying an ElivisionTM plus Polyer HP Kit (Maixin Biotechnologies, Inc., Fuzhou, China). Freshly cut slides (4-μm thick) underwent deparaffinage, hydration, blockage of endogenous peroxidase activity with 3% hydrogen peroxide, and heat mediated antigen retrieval. The sections were then incubated with anti-HMGA1 (ab129153; 1:100 dilution; Abcam, United States) and anti-MAFG (ab154318; 1:100 dilution; Abcam) antibodies at 4°C overnight, followed by incubation with a polymer helper reagent and poly-peroxidase-anti-mouse/rabbit IgG. Finally, the sections underwent diaminobenzidine (Maixin Biotechnologies, Inc.,) staining, dehydration, and mount. Additionally, the protein expressions of TFs in the risk score in HCC and normal hepatocellular tissues were obtained from the Human Protein Atlas (HPA, https://www.ptroteinatlas.org/) which provided the IHC data of human tissues (Uhlén et al., 2015). The staining intensity was scored as 0 (negative staining), 1 (weak staining), 2 (moderate staining) or 3 (strong staining). The extent of positive staining was scored as 1 (<25%), 2 (25%–75%), 3 (>75%). The final score was obtained by multiplying the intensity and extent scores.

### Statistical analysis

All the statistic work was accomplished by the R software (version 4.1.2). Overall survival (OS) was calculated by the Kaplan-Meier method, with the difference between groups compared using the log-rank test. The paired *t*-test was used to compare the transcriptional and protein differences in HCC and paired non-tumorous tissues in our cohort. The comparison of other continuous data was performed by the Wilcoxon test between two groups and Kruskal–Wallis test among three or more groups. A *p*-value < 0.05 was considered statistically significant.

## Results

### Identification of prognostic DETFs in HCC

**FIGURE 1:**
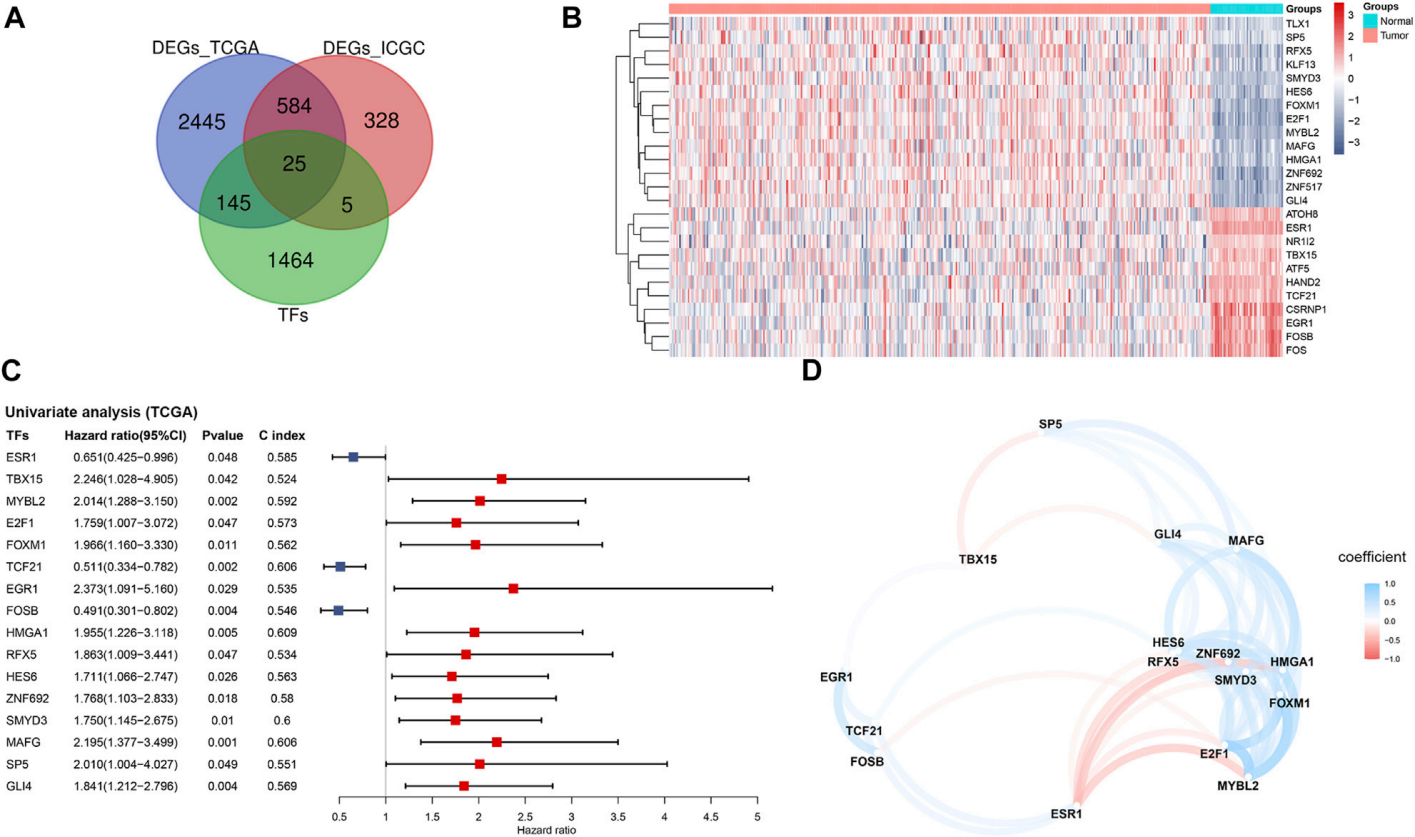
Identification of prognosis-related TFs in HCC based on the TCGA cohort **(A)** Venn diagram of differentially expressed TFs between tumor and adjacent normal tissues shared in the TCGA and ICGC cohorts **(B)** Heatmap of the 25 overlapping TFs in the TCGA cohort **(C)** Forest plot of the prognosis-related TFs based on univariate Cox regression analysis **(D)** The correlation network of prognosis-related TFs. TFs, transcription factors; TCGA, The Cancer Genome Atlas; ICGC, International Cancer Genome Consortium.

Based on the TCGA and ICGC cohorts, a total of 3,119 and 942 DEGs were identified respectively in tumor tissues compared with the respective non-tumorous tissues. Among the overlapping differentially expressed ones in the TCGA and ICGC cohorts, 25 were TFs (Figure 1A). According to the expression profile (Figure 1B), 14 DETFs were upregulated and 11 downregulated in tumor tissues in the TCGA cohort. The results of univariate analysis demonstrated that 16 of the 25 DETFs were associated with the prognosis of patients. ESR1, TCF21 and FOSB, which were downregulated in HCC, predicted a favorable outcome. The other two downregulated ones and 11 upregulated TFs in HCC indicated an adverse prognosis (Figure 1C). According to the correlation network, most of the TFs were positively related, except for TBX15, ESR1 and FOSB, which had negative correlations with some TFs (Figure 1D).

### Construction and validation of a 2-TF-based prognostic signature

**FIGURE 2:**
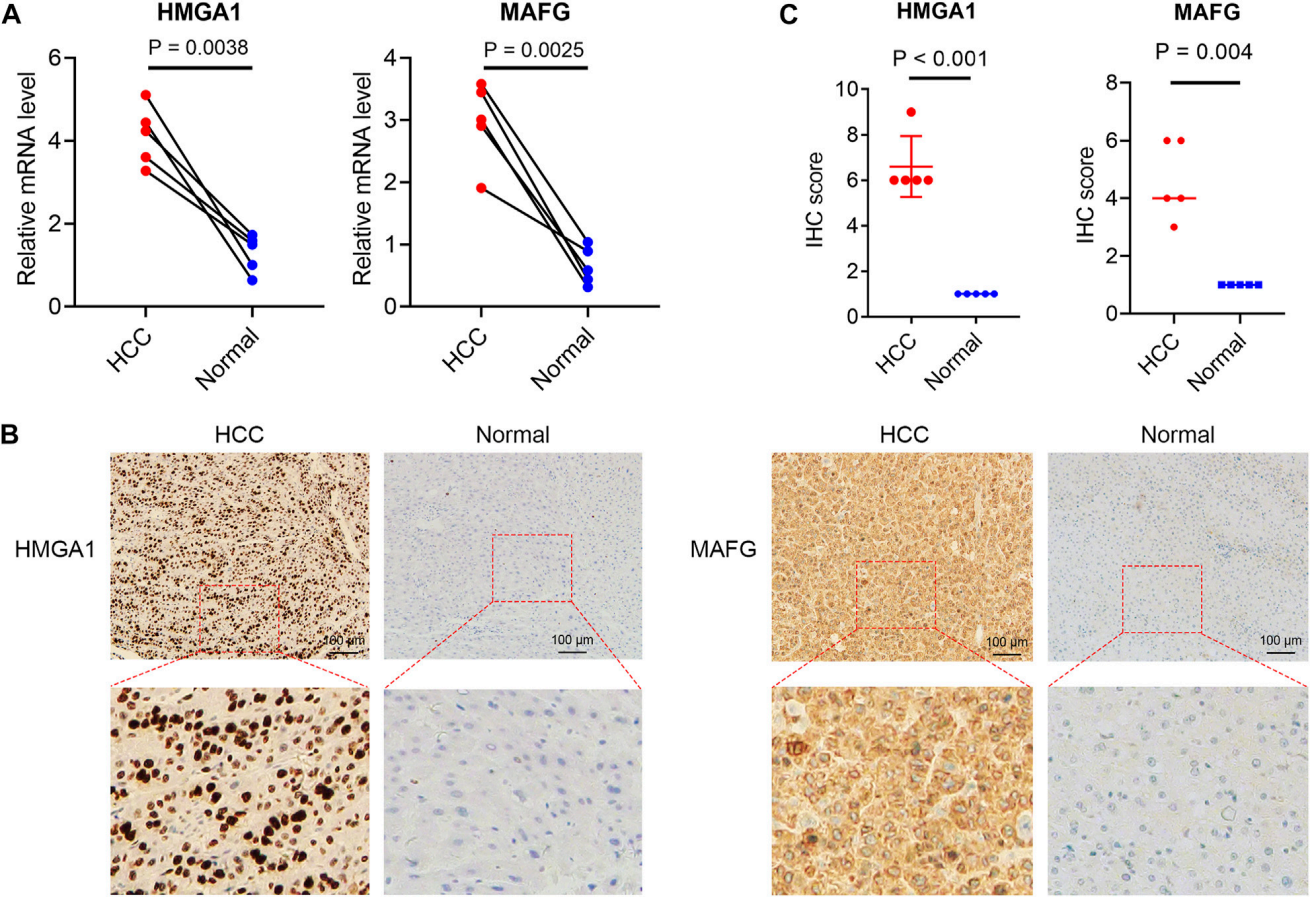
Validated expressions of TFs in the TF signature in patient samples **(A)** The mRNA levels of HMGA1 and MAFG in tumor and adjacent non-tumorous tissues from our cohort. The immunohistochemical staining **(B)** and statistical significance **(C)** of HMGA1 and MAFG in HCC and normal liver tissues from our cohort. TFs, transcription factors.

**FIGURE 3:**
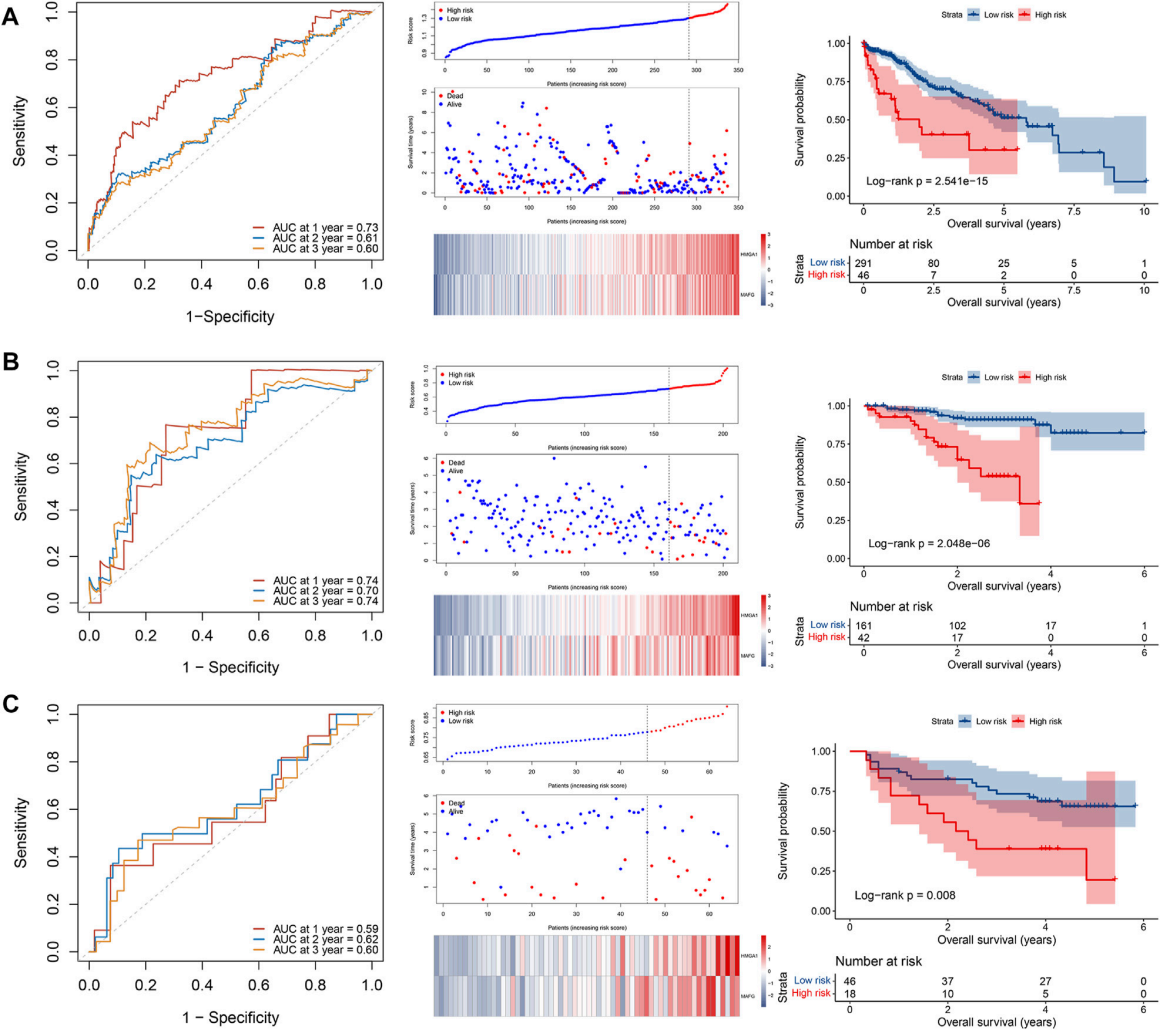
Construction and validation of a 2-TF prognostic signature. The ROC curve, distributions of the risk score and survival status, heatmap of expression profiles, and Kaplan–Meier curve of the 2-TF signature in the training set of TCGA cohort **(A)**, as well as in the validation sets of ICGC cohort **(B)** and GSE116174 cohort **(C)**. TF, transcription factor; AUC, area under the curve; ROC, receiver operating characteristic; TCGA, The Cancer Genome Atlas; ICGC, International Cancer Genome Consortium.

The 16 prognosis-related TFs were included to establish a prognostic model in the training set of TCGA cohort *via* LASSO Cox regression analysis. A 2-TF-based signature, comprising high mobility group AT-hook protein 1 (HMGA1) and MAF BZIP transcription factor G (MAFG), was identified based on the optimal value of *λ* (Supplementary Figure S1). The transcriptional (Figure 2A) and protein (Figures 2B, C) levels of HMGA1 and MAFG were validated to be elevated in HCC specimens compared to those in adjacent normal tissues in our retrospective cohort. High or medium staining intensity of protein expression of the two TFs by IHC was also observed in HCC tissues based on the HPA database (Supplementary Figures S2A, B). The insignificant difference of MAFG expression between tumor and normal tissues (Supplementary Figure S2B) may be attributed to the antibody specificity. The risk score = 0.0924 × HMGA1 + 0.0658 × MAFG. Then, the risk score for each patient was determined accordingly in the TCGA cohort as well as in the validation sets of ICGC and GSE116174 cohorts. The AUCs for the 2-TF signature were 0.73 for 1-year, 0.61 for 2-year, and 0.60 for 3-year OS in the TCGA cohort (Figure 3A). In the validation sets, the AUCs were 0.74, 0.70, 0.74 in the ICGC cohort (Figure 3B), and 0.59, 0.62, 0.60 in the GSE116174 cohort for 1-, 2- and 3-year OS, respectively (Figure 3C). A relative low predictive efficiency of the TF signature in the GSE116174 cohort may be attributed to a small sample size. The patients were then divided into high- or low-risk group based on the optimal cut-off. As the risk score increased, the number of deaths increased and the survival time decreased, with the expressions of the 2 TFs also in an increased trend (Figures 3A–C). According to the Kaplan-Meier curve, patients in the high-risk group suffered a significantly more unfavorable OS than their low-risk counterparts in the TCGA cohort as well as in the ICGC and GSE116174 cohorts (Figures 3A–C). Collectively, our results indicated that the 2-TF signature had a good performance for predicting the OS of HCC patients.

### Independent prognostic role of the 2-TF signature

**FIGURE 4:**
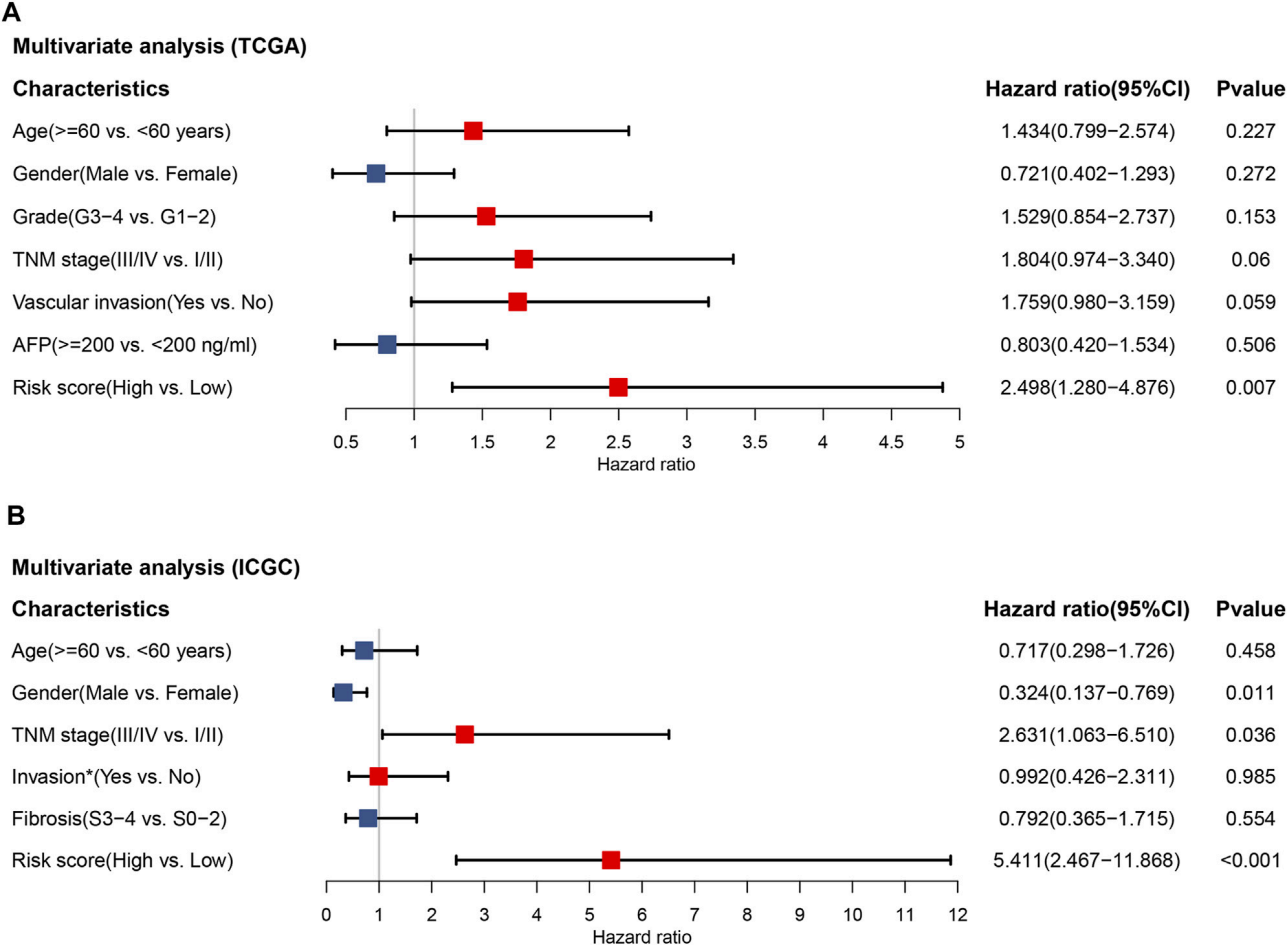
The independent prognostic value of the 2-TF signature. Multivariate Cox regression analysis regarding overall survival in the training set of TCGA cohort **(A)** and the validation set of ICGC cohort **(B)**. * Portal vein, vein or artery invasion; TCGA, The Cancer Genome Atlas; ICGC, International Cancer Genome Consortium.

In the TCGA cohort, 245 patients with complete information including age, gender, tumor grade, TNM stage, vascular invasion and alpha-fetoprotein (AFP) were included for multivariate Cox regression analysis. The results demonstrated that the risk score was the only factor significantly associated with OS in the TCGA cohort (HR = 2.498, 95% CI = 1.280–4.876, *p* = 0.007) (Figure 4A). To validate our results, 203 patients in the ICGC cohort with age, gender, TNM stage, invasion (portal vein, vein or artery) and fibrosis underwent multivariate analysis. As shown in Figure 4B, the risk score was still capable of independently predicting OS (HR = 5.411, 95% CI = 2.467–11.868, *p* < 0.001). Therefore, the 2-TF signature could be an independent indicator for an adverse outcome in HCC patients.

### Relationships between the 2-TF signature and clinicopathological characteristics

**FIGURE 5:**
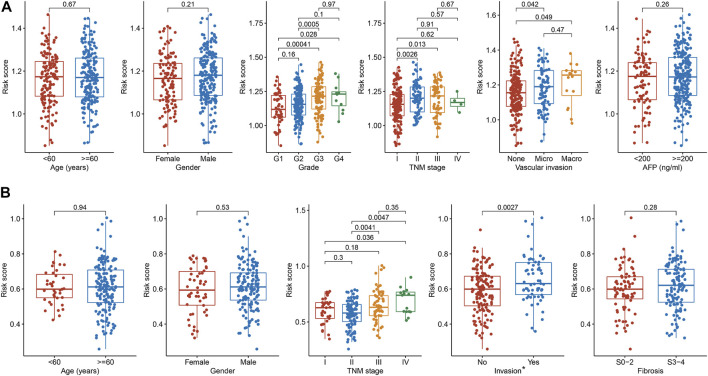
The relationships between the TF signature and clinical indicators **(A)** The correlations of risk score with age, gender, grade, TNM stage, vascular invasion and AFP level in the TCGA cohort **(B)** The correlations of risk score with age, gender, TNM stage, invasion and fibrosis in the ICGC cohort. * Portal vein, vein or artery invasion; TF, transcription factor; AFP, alpha-fetoprotein; TCGA, The Cancer Genome Atlas; ICGC, International Cancer Genome Consortium.

To comprehensively evaluate the involvement of the 2-TF signature in the tumorigenesis of HCC, we further explored the associations of the risk score with clinicopathological features. In the TCGA cohort (Figure 5A), G3 or G4 tumors tended to have a higher risk score compared to G1 tumors. The risk score between G2 and G3 tumors was also significantly different. Regarding TNM stage, the risk score increased as the stage I increased to stage II or III. For patients with vascular invasion, either micro- or macro-invasion cases, the risk score was higher compared to those without invasion. In the ICGC cohort (Figure 5B), similar relationship of the risk score with TNM stage and invasion were observed. There was no significant correlation between the TF signature and age, gender, fibrosis or AFP level of HCC in the cohorts. Therefore, the 2-TF signature was involved in the tumor grade, TNM stage and tumor invasion in HCC.

### Evaluation of treatment efficacy based on the 2-TF signature

**FIGURE 6:**
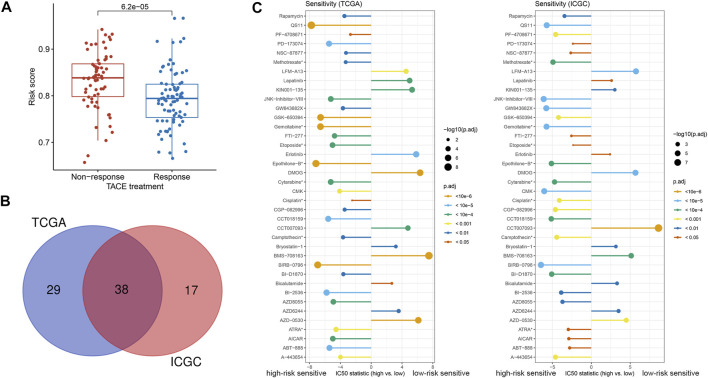
Treatment efficacy based on the 2-TF signature **(A)** The correlation between response to TACE treatment and risk score in GSE104580 **(B)** The common agents estimated to have different IC_50_ according to the risk score both in the TCGA and ICGC cohorts **(C)** The discrepancy of estimated IC_50_ in the high- and low-risk groups in the TCGA and ICGC cohorts. *Chemotherapeutic agents; TF, transcription factor; IC_50_: half-maximal inhibitory concentration; TACE: transarterial chemoembolization; TCGA, The Cancer Genome Atlas; ICGC, International Cancer Genome Consortium.

According to the guideline, TACE is a standard treatment for intermediate-stage HCC (Chang et al., 2020). The clinical treatment cohort of GSE104580 was applied to investigate the association of the 2-TF signature with TACE treatment benefit. The results demonstrated that patients with a low-risk score had a desirable efficacy for TACE treatment (Figure 6A). Using the pRRophetic algorithm, we further evaluated the IC_50_ of numerous agents to determine latent therapeutic drugs. There were 67 and 55 agents associated with the risk score in the TCGA and ICGC cohorts respectively, 38 of which were common between them (Figure 6B). The IC_50_ difference of the 38 drugs was displayed in Figure 6C (all adjusted *p* < 0.05). Our results indicated that the high-risk patients tended to be more sensitive to numerous targeted agents, such as ABT-888 (veliparib, a PARP inhibitor), AZD8055 and rapamycin (both mTOR inhibitors), as well as the eight common chemotherapeutic agents, including ATRA, camptothecin, cisplatin, cytarabine, epothilone-B, etoposide, gemcitabine and methotrexate. Whereas patients in the low-risk group were prone to lapatinib, erlotinib and other targeted agents (Figure 6C). Based on our results, combined treatments of TACE with these drugs might be promising therapeutic regimens if administrated according to the classified subtypes of HCC patients.

### Molecular characteristics and pathways of the 2-TF signature

**FIGURE 7:**
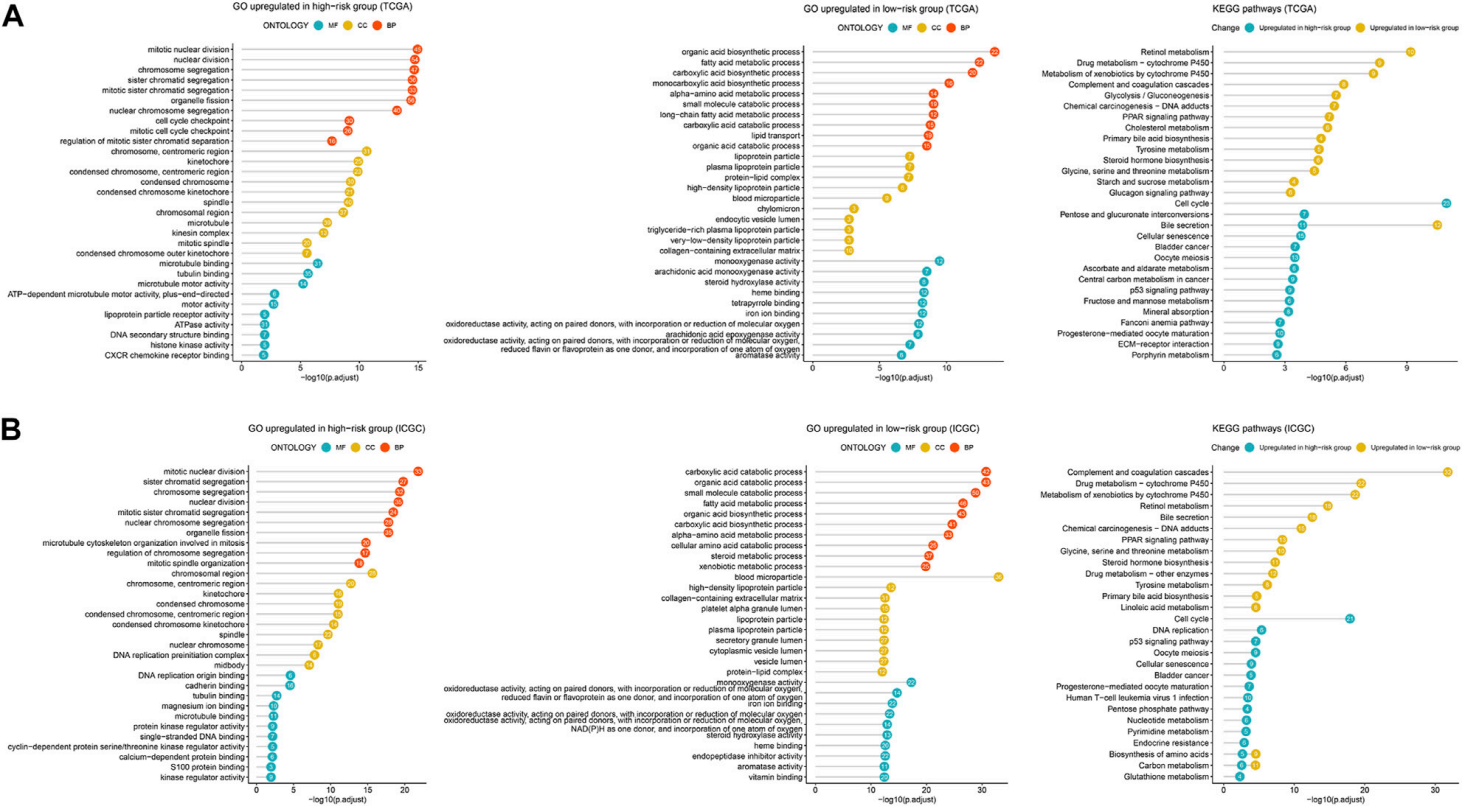
Enrichment analysis of DEGs related to the 2-TF signature. The most significant GO terms and KEGG pathways based on the DEGs between the high- and low-risk groups in the TCGA cohort **(A)** and ICGC cohort **(B)**. DEGs, differentially expressed genes; GO, Gene Ontology; KEGG, Kyoto Encyclopedia of Genes and Genomes; TCGA, The Cancer Genome Atlas; ICGC, International Cancer Genome Consortium.

To uncover the biological characteristics influenced by the risk score, the DEGs between the high- and low-risk groups in the TCGA and ICGC cohorts were identified and underwent GO enrichment and KEGG pathway analyses. According to the most significant GO terms as shown in Figure 7, upregulated genes in the high-risk group both in the TCGA (Figure 7A) and ICGC (Figure 7B) cohorts were mainly enriched in cell proliferation-related functions, such as nuclear division and chromosome segregation. Similarly, the top KEGG pathways related to the upregulated genes in the high-risk group were cell cycle, cellular senescence and oocyte meiosis in both cohorts (Figures 7A, B). The genes upregulated in the low-risk group were enriched in various biosynthetic and metabolic processes both in the TCGA and ICGC cohorts, such as fatty acid metabolic process, alpha-amino acid metabolic process, carboxylic acid biosynthetic and catabolic processes, and organic acid biosynthetic and catabolic processes (Figures 7A, B). Consistently, according to the KEGG pathways, upregulated genes in the low-risk group were preferentially related to retinol metabolism, cytochrome P450-related metabolism, complement/coagulation cascades and peroxisome proliferator-activated receptor (PPAR) signaling (Figures 7A, B). Therefore, the 2-TF signature divided the patients into proliferative and metabolic subtypes with different biological processes, which may explain the prognostic prediction performance.

### Pivotal genes related to the 2-TF signature

**FIGURE 8:**
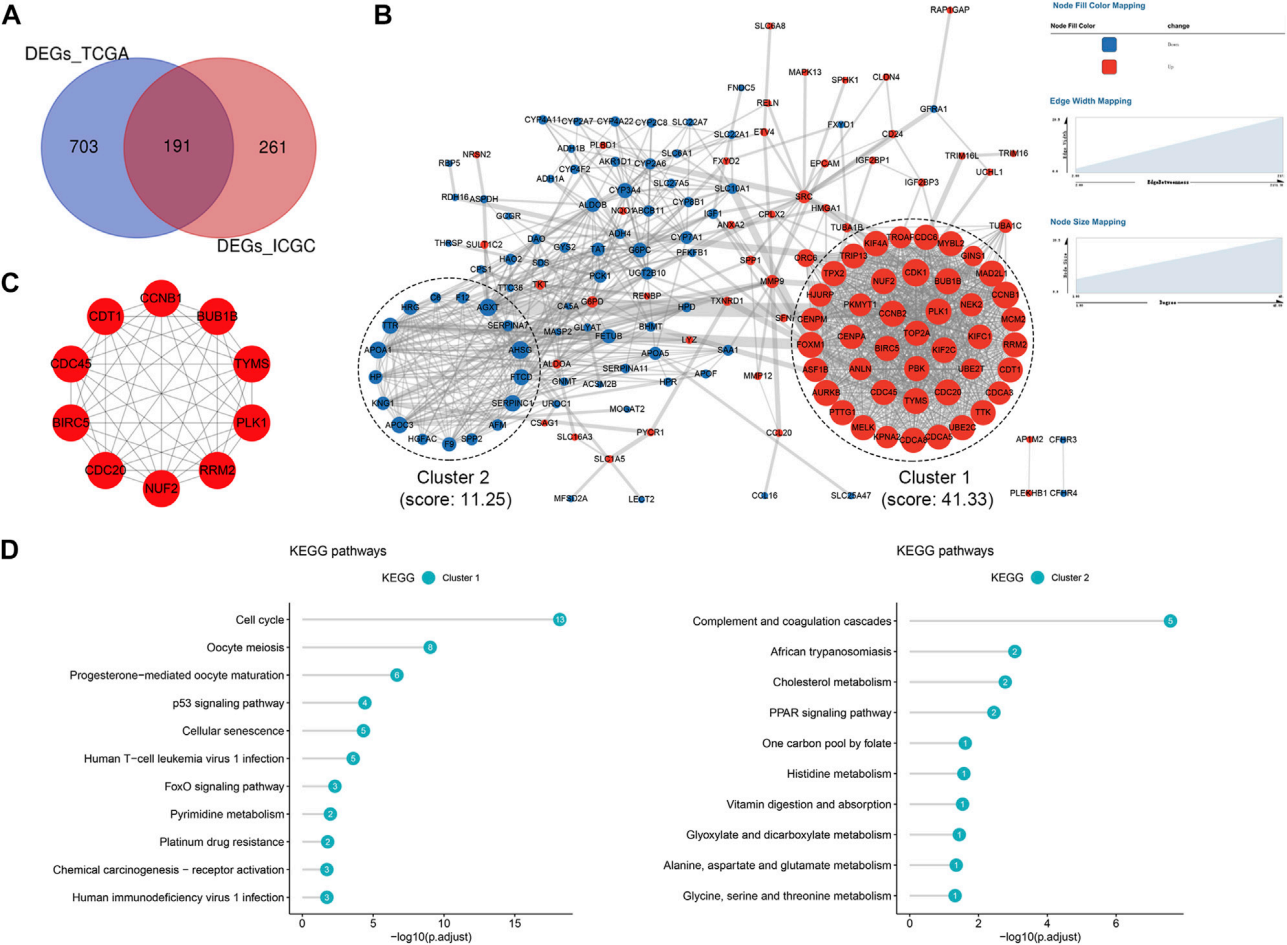
Protein-protein interaction (PPI) network based on the DEGs **(A)** Venn diagram of DEGs between the high- and low-risk groups shared in the TCGA and ICGC cohorts **(B)** PPI network constructed with the common DEGs and functional modules **(C)** The top 10 hub genes identified based on the PPI network **(D)** KEGG pathway enrichment analysis of module one and module 2. DEGs, differentially expressed genes; TCGA, The Cancer Genome Atlas; ICGC, International Cancer Genome Consortium.

There were 191 common DEGs between the high- and low-risk groups in the TCGA and ICGC cohorts (Figure 8A). Subsequently, a PPI network containing 164 nodes and 1,358 interactions was established based on the 191 DEGs. Additionally, two significant modules with a score ≥5, namely module 1 comprising 43 upregulated genes in the high-risk group and module 2 comprising 17 upregulated genes in the low-risk group, were identified *via* MCODE (Figure 8B). The top 10 hub genes were further screened out, including BUB1B, TYMS, PLK1, RRM2, NUF2, CDC20, BIRC5, CDC45, CDT1 and CCNB1, which were all included in module 1 (Figure 8C). All the hub genes were validated to be closely correlated in HCC based on the TCGA, ICGC and GSE116174 cohorts (Supplementary Figure S3A–C). Enrichment analysis demonstrated that the most significant pathway in module 1 were cell cycle, and module 2 were mainly enriched in complement and coagulation cascades pathway (Figure 8D).

## Discussion

Hepatocellular carcinoma (HCC) is one of the deadliest human health burdens in the world, with adverse outcome and limited treatment strategies. High recurrent incidence of tumor and poor response to therapeutic agents are the primary attributors for the unfavorable prognosis (Venook et al., 2010). Therefore, it is of urgency to identify more valuable makers to predict the prognosis and therapeutic efficacy for HCC patients. Recent studies highlighted the roles of transcription factors in cancer which were validated to participant in carcinogenesis in terms of cell proliferation, invasion, metastasis and patient survival (Bushweller 2019). In this study, we screened out 16 prognosis-related TFs in HCC and identified a 2-TF signature as an independent prognostic indicator for patients *via* multiple public cohorts from TCGA, ICGC and GEO databases. In addition, the 2-TF signature was significantly positively associated with progressive characteristics regarding tumor grade, TNM stage and tumor invasion. Patients with a high-risk score tended to be resistant to treatments of TACE, lapatinib and erlotinib, but sensitive to chemotherapeutic agents. Further analysis demonstrated that the tumors hold proliferative or metabolic features according to the 2-TF signature, which may explain the diversities regarding prognosis and treatment response in HCC patients.

The TF-based signature identified in this study was comprised of HMGA1 and MAFG, whose combination was not reported in previous studies. HMGA1 is a non-histone chromatin protein responsible for chromatin remodeling to regulate gene transcription (Sumter et al., 2016). Although previously be regarded to be rarely expressed in adult tissue (Chiappetta et al., 1996), HMGA1 has been observed to be overexpressed in various malignant cells in recent studies (Wang et al., 2019). HMGA1 also drives neoplastic transformation, anti-apoptosis, angiogenesis as well as immune evasion in cancer (Sumter et al., 2016). HCC is commonly secondary to chronic infection with HBV and subsequent liver cirrhosis. According to the study by Shen et al., HMGA1 protein could positively regulate HBV transcription *via* recruiting transcription factors forkhead box O3alpha (FOXO3α) and peroxisome proliferator-activated receptor-γ coactivator-1α (PGC1α). On the other hand, HMGA1-targeted treatment facilitates HBV clearance (Shen et al., 2022). Similarly, Andreozzi et al. observed monotonically increased HMGA1 expression in tissues from normal liver to cirrhotic transformation, to primary HCC and to disease metastasis. Additionally, HCC patients with positive HMGA1 expression had a high Edmondson grade, shorter disease-free and overall survival (Andreozzi et al., 2016). Consistently, our study found increased mRNA and protein levels of HMGA1, which was associated with worse overall survival of HCC patients. Additionally, a high score of the HMGA1-included risk model could predict an advanced tumor grade, TNM stage, tumor invasion, and dismal clinical outcome for HCC patients. There is another HMGA1-involved signature been proved perform well in distinguishing HCC patients at a high risk of tumor recurrence (Son et al., 2022). In gastric cancer, HMGA1 overexpression enhances the migration and invasion abilities and promotes epithelial-to-mesenchymal transition of cancer cells (Ouyang et al., 2022). HMGA1 activation also increases the cell viability and migration of liver cancer cells, indicating its driving role in liver carcinogenesis (Andreozzi et al., 2016).

MAFG, as a member of the basic leucine zipper family of transcription factors, is a small MAF protein. MAFG is essential for the transcription of nuclear factor-erythroid 2 related factor 2 (NRF2), since NRF2 binds the antioxidant response element (ARE) *via* MAFG-NRF2 heterodimer to activate antioxidant genes, mediating stress response and detoxification (Kannan et al., 2012). A recent study by Liu et al. observed MAFG overexpression in human HCC tissues, which was correlated with hepatitis B, tumor grade, vascular invasion, and worse prognosis of patients (Liu et al., 2018). Our study confirmed MAFG overexpression both in mRNA and protein levels in HCC, and its promoting role for advanced tumor grade, TNM stage, tumor invasion and dismal prognosis in HCC patients. A previous study reported the contribution of lncRNA MAFG-AS1 to the cell proliferation and migration of HCC *via* activating MAFG transcription (Zhang et al., 2021). Upregulated MAFG-AS1 with copy amplification was also associated with the dismal prognosis of HCC (Du et al., 2020). On the contrary, functional silencing of MAFG could repress the liver homing of tumor cells in colorectal cancer, indicating its promoting roles in metastasis and liver colonization in colorectal cancer (Torres et al., 2018). There were contradictory views regarding the contributions of age, gender and cirrhosis to patient survival in HCC, excepting AFP which was an unfavorable factor (Kitisin et al., 2011; Polychronidis et al., 2022; Son et al., 2022). The current study found no relationship of the HMGA1-MAFG signature with age, gender, fibrosis and AFP in HCC patients. Additionally, with the adjustment of those clinicopathological characteristics, a high 2-TF risk score indicated adverse survival in HCC. We supposed that the HMGA1-MAFG signature may contribute to the clinical outcome of HCC patients independent of those factors. The underlying mechanism is warranted to be uncovered by biology experiments in the future.

To understand the prognostic significance of the 2-TF signature, our study further delineated the underlying biological features of tumors in the high- and low-risk groups. According to the results of GO and KEGG analyses, high-group patients were a proliferative subtype which was mostly enriched in nuclear division, cell cycle and cellular senescence. Whereas the low-risk patients were mainly a metabolic subtype which was correlated with oxidoreductase activity, cytochrome P450-induced metabolism and numerous endogenous metabolism. Our study was consistent with the previous studies (Chen et al., 2020) (Liu et al., 2022). The study by Liu et al. identified three heterogeneous clusters based on immune cell evolutions, with proliferative HCC a more advanced pathological stage and dismal prognosis relative to lipid metabolic and immune inflammatory tumors (Liu et al., 2022). Another study by Chen et al. divided HCC according to energy metabolism genes, observing a positive correlation of a metabolism-enriched subtype with better survival (Chen et al., 2020). Upregulated HMGA1 was reported to be involved in DNA replication, cell cycle and several proliferation-related signaling pathways, and the downregulated HMGA1 was enriched in tryptophan metabolism, fatty acid metabolism, primary bile acid biosynthesis and PPAR signaling pathway (Wei et al., 2022). According to Tian et al., the activation of HMGA1/STAT3 pathway promotes the proliferation, migration and invasion of HCC cells (Tian et al., 2021). Downregulation of HMGA1 dramatically suppresses HCC proliferation *via* inducing cell cycle arrest (Shi et al., 2022). HMGA1 can also induce liver metastasis *via* promoting glucose transporter 3 (GLUT3) transcription and expression in CRC cells (Yang et al., 2020). Since GLUT3 favors the glucose transport into tumor cells to satisfy their high metabolism and rapid growth. Retinol-binding protein (RBP4)-related disruption of retinol metabolism was reported to participant in malignant tumors (Papiernik et al., 2020). HMGA1 plays an important role in the transcription of RBP4 (Bianconcini et al., 2009). Similar to HMGA1, NRF2/MAFG heterodimer promotes the cell proliferation in HCC (Pan et al., 2022). On the other hand, as a target of farnesoid X receptor (FXR), hepatic MAFG overexpression represses bile acid synthesis and metabolism (de Aguiar Vallim et al., 2015). Collectively, energy metabolism and proliferative characteristics based on the 2-TF signature could uncover tumor heterogeneity with distinct clinical significance in HCC.

Since the TACE treatment is recommended for primary HCC patients (Chang et al., 2020). We further explored the association of the risk model with TACE treatment response and found that patients with a high risk score tended to suffer resistance. Subsequently, individual drugs demonstrating distinct effects between patients with different molecular features were uncovered. Tumors with proliferative characteristics were more sensitive to conventional chemotherapeutics, whereas those with metabolic characteristics were susceptible to tyrosine kinase inhibitors such as lapatinib and erlotinib. Our results were consistent with the study by Liu et al., where they identified obatoclax, docetaxel, and cisplatin as potential therapeutic agents for tumors in proliferative clusters, whereas recommended nilotinib and bosutinib for tumors in lipid metabolic clusters (Liu et al., 2022). According to the study by Li et al., HMGA1 promotes the proliferation of liver cancer *via* regulating the Akt signaling pathway (Liu et al., 2017). Akt acts as a crucial mediator of growth factor-induced cell proliferation *via* the activation of mammalian target of rapamycin (mTOR) (Revathidevi and Munirajan 2019). Silencing MAFG in hepatocytes improves glucose metabolism and impairs mTOR activation (Pradas-Juni et al., 2020). Likewise, our results observed increased sensitivity of HCC in the high-risk group to the mTOR inhibitors, AZD8055 and rapamycin. Additionally, an increased interaction between MAFG and NRF2 was previously observed upon tumor exposure to the kinase inhibitor of sorafenib, whereas NRF2 inhibition promoted the response to sorafenib in HCC (Sun et al., 2016). Therefore, the 2-TF signature possibly indicates tumor sensitivity to sorafenib in HCC, which requires further validation. Overall, our TF-based signature may provide clues for individualized clinical management according to distinct molecular features, thus improving the therapeutic efficacy for HCC.

This study is advantaged at the identification and validation of a novel 2-TF signature consisted of HMGA1 and MAFG, which is able to predict the prognosis and therapeutic response of HCC *via* distinguishing the characteristics of tumor proliferation and metabolism. However, there is some limitations. The expressions of HMGA1 and MAFG were only validated in HCC patients, which require further verification in cultured HCC cells. Besides, biology assay is needed to identify the proliferative and metabolism pathways and downstream targets susceptible to the two TFs, so as to uncover the underlying mechanism of the TF signature to the disease progression and patient survival in HCC. Last, more preclinical studies and prospective clinical trials are warranted to confirm our findings.

## Conclusion

In conclusion, the present study identified a TF-based risk model consisted of HMGA1 and MAFG in HCC based on public datasets from multiple databases. The risk model distinguished progressive characteristics regarding tumor grade, TNM stage and tumor invasion, and showed independent predictive capability for adverse prognosis in HCC. Additionally, the risk model decoded tumor heterogeneity with proliferative and metabolic features, with the former sensitive to chemotherapy and the latter recommended for TACE plus tyrosine kinase inhibitors of lapatinib and erlotinib. Our study provides potential prognostic biomarkers and corresponding individualized treatment strategies, which aid optimal clinical management and improved clinical outcome for HCC patients.

## Data Availability

The original contributions presented in the study are included in the article/Supplementary Material, further inquiries can be directed to the corresponding authors.
