# Corrigendum: A Novel transcription factor-based signature to predict prognosis and therapeutic response of hepatocellular carcinoma

**DOI:** 10.3389/fgene.2023.1146199

**Published:** 2023-01-25

**Authors:** Yanbing Yang, Xuenian Ye, Haibin Zhang, Zhaowang Lin, Min Fang, Jian Wang, Yuyan Yu, Xuwen Hua, Hongxuan Huang, Weifeng Xu, Ling Liu, Zhan Lin

**Affiliations:** ^1^ Department of Radiology, Mengchao Hepatobiliary Hospital of Fujian Medical University, Fuzhou, China; ^2^ Department of Orthopedics, Dongguan People’s Hospital, Dongguan, China; ^3^ Department of Medical Oncology, The Affiliated Cancer Hospital of Zhengzhou University, Zhengzhou, China; ^4^ Department of Radiology, The First Affiliated Hospital of Dali University, Dali, China

**Keywords:** transcription factor, high mobility group AT-hook protein 1, MAF BZIP transcription factor G, prognosis, therapeutic response, hepatocellular carcinoma

In the original article, there was a mistake in the **Funding** statement as published. The correct grant number for the Startup Fund for Scientific Research of Fujian Medical University is 2018QH1202. Corrected funding statement is given below:

“This work was supported by the Scientific Research Project for the Middle-aged and Youths of Fuzhou Health Department (grant number: 2019-S-wq9) and the Startup Fund for Scientific Research of Fujian Medical University (grant number: 2018QH1202).”

The authors apologize for this error and state that this does not change the scientific conclusions of the article in any way. The original article has been updated.

