# Supplementary figures and images for: A novel transcription factor-based signature to predict prognosis and therapeutic response of hepatocellular carcinoma
Source: Front Genet. 2023 Jan 4;13:1068837. doi: 10.3389/fgene.2022.1068837 (PMC9845592; doi:10.3389/fgene.2022.1068837)

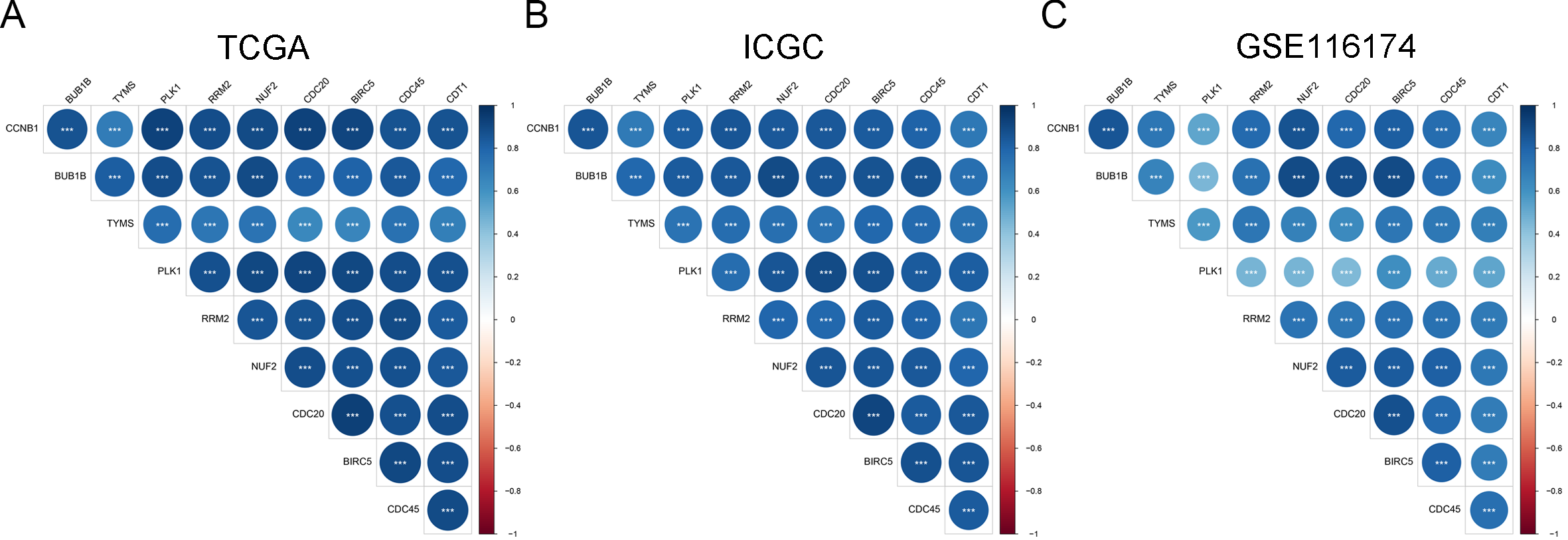

Supplement: Supplementary file 1 [file Image3.TIF]

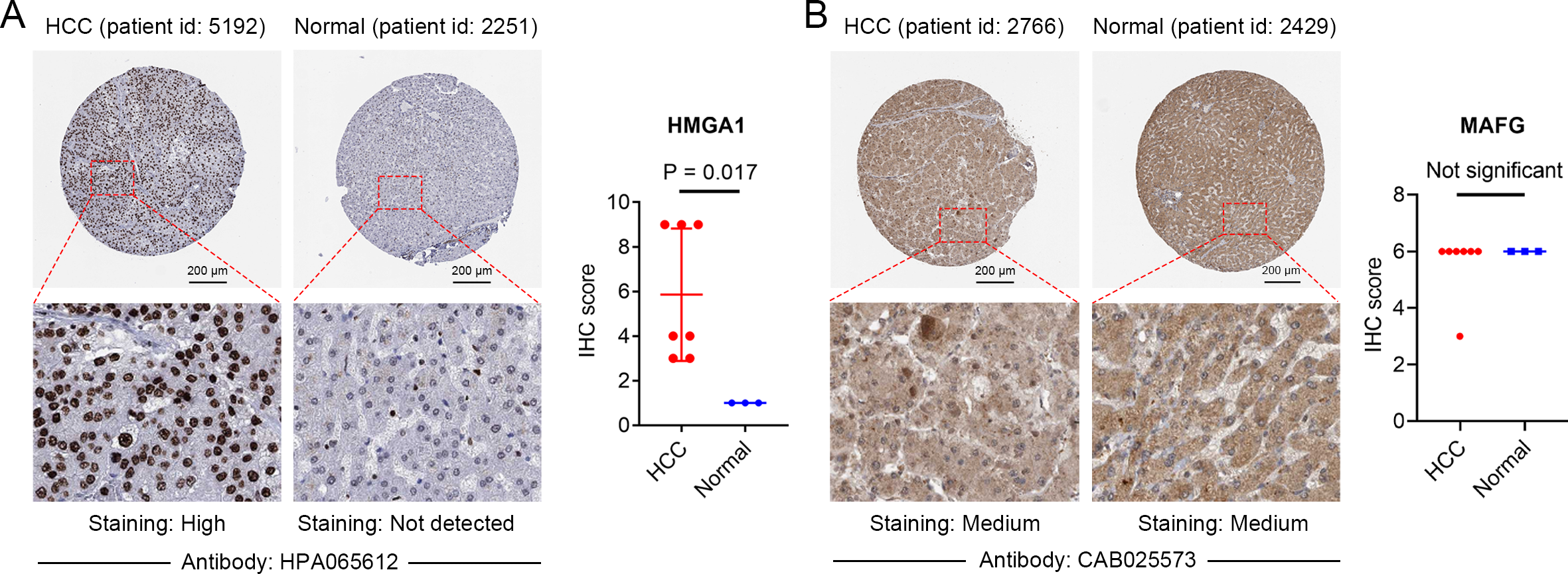

Supplement: Supplementary file 2 [file Image2.TIF]

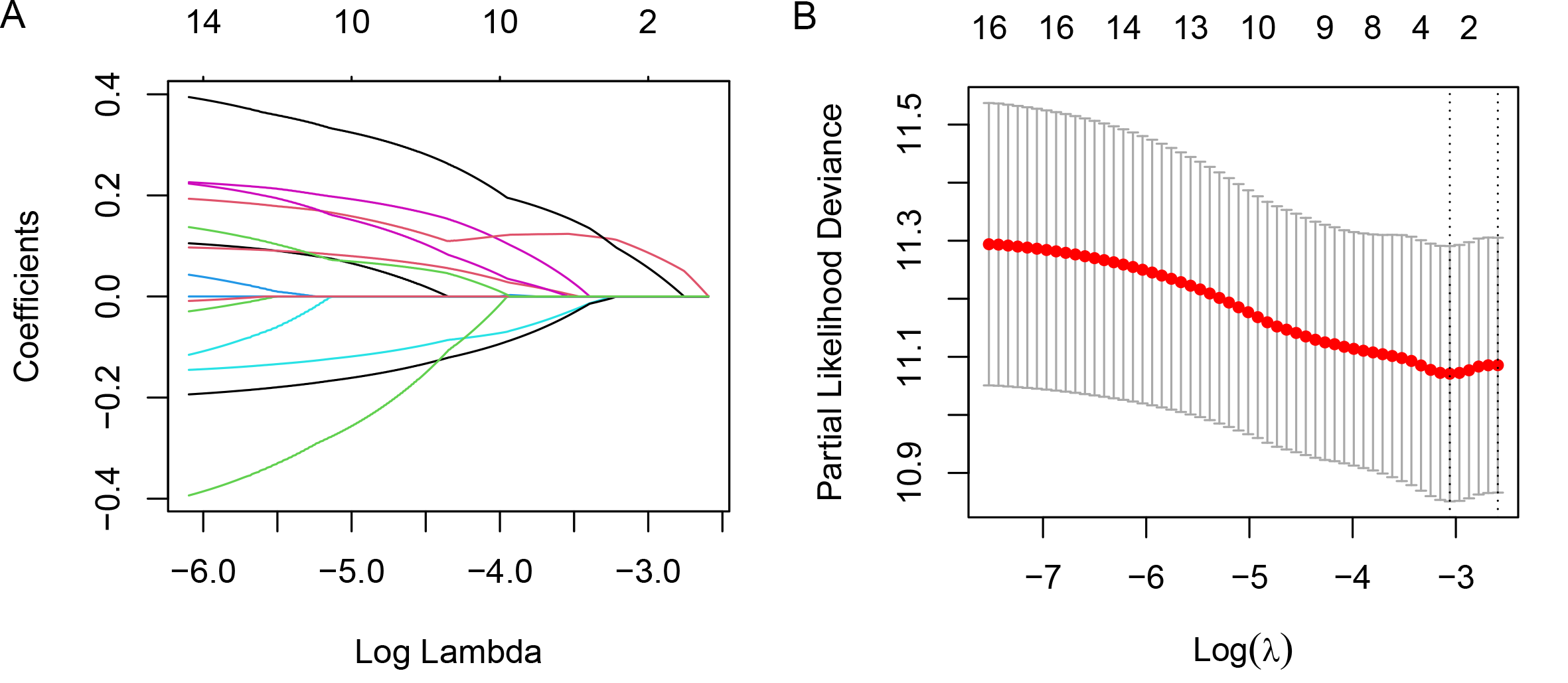

Supplement: Supplementary file 3 [file Image1.TIF]
